# The Next Disease X‐ H5N6 Avian Influenza's Evolving Threat to Human Health and Chances of Future Global Outbreak: A Narrative Review

**DOI:** 10.1002/hsr2.71526

**Published:** 2025-11-17

**Authors:** Shoubeho Sadique Shandhi, Mosiur Rahman, Mohammad Shahriar, Ramisa Anjum

**Affiliations:** ^1^ Department of Pharmacy University of Asia Pacific Farmgate 1205 Dhaka Bangladesh

## Abstract

**Background and Aims:**

H5N6 avian influenza has been a concern that can initiate the next global pandemic (called Disease X). Most infections have been among those in Asia, especially China, since the 2014 first human case. With a fatality rate of 61%, the virus is very deadly, even though the total number of human cases is relatively low. This paper aims to evaluate the epidemiological trends, genetic evolution, and zoonotic potential of H5N6, with a focus on its capacity to become a future pandemic agent.

**Methods:**

This narrative review is based on a comprehensive synthesis of published literature, including peer‐reviewed epidemiological studies, genetic analyses and clinical case reports. Key themes examined include viral evolution, host adaptation, transmission patterns, and disease pathogenesis, with particular attention to the zoonotic potential and public health implications of H5N6.

**Results:**

Genetically, H5N6 is still evolving and is moreover becoming increasingly adept at infecting birds and mammals. The epidemiology of the virus, its genetic mutations, and its patterns of transmission are examined with emphasis on how the virus can cross from animals to humans. H5N6, however, has demonstrated the capacity to bind to human respiratory cells, making this a potentially more likely vector of human‐to‐human transmission in the future. The virus is evolving, however, and while sustained transmission between people has not yet taken place, the developing risk is expanding.

**Conclusion:**

The only thing that should help here is that global health systems must be more vigilant by adding stronger bio‐security measures, more surveillance, and more common efforts toward vaccine research. The spread of H5N6 in both domestic and wild populations also means that a perpetually high level of monitoring, especially in high‐risk areas such as live poultry markets, is necessary. H5N6 could evolve to become a serious public health threat and this paper highlights the urgent need for preparedness.

## 1 Background

The name Disease X is given to an unknown pathogen that could later trigger a global pandemic. It refers to things a public health system needs to be defended against, from a brand‐new virus to an existing one that mutates into a worse virus. WHO defined this term to indicate the necessity for forward‐thinking readiness [1]. By focusing on viruses that can lead to a pandemic and accelerating the development of vaccines and diagnostics, Disease X helps get ready for whatever could happen next [2]. The emergence of an unknown pathogen that can cause a global pandemic is represented with the idea of “Disease X”. It is a warning that new diseases, especially those that can jump from animals to humans are always a possibility [3]. This is where the idea of Disease X comes in, a concept that means public health systems need to be proactive and stay ahead, not playing catch up once it's too late. Tools such as ‘SpillOver’ (from the University of California, Davis) highlight for example which pathogens are at the highest risk of becoming the cause of a pandemic and need to be monitored closely [4]. Thus, the focus on Disease X helps speed vaccine development, better diagnostics, and global cooperation so we are ready for whatever comes next [3, 4]. More than ever, the world must be prepared to deal with new and unpredictable health threats. Disease X is no imaginary scenario it's an intense reminder to remain sharp and prepared in case something does happen. Hence, it becomes an incentive to develop better tools and strategies for fighting infectious diseases before they can transform into full‐blown global crises [5].

Influenza A virus (IAV) is a very common virus that can cause serious illness in humans. It mainly lives in wild birds but can also infect many animals like poultry, seals, dolphins, cats, dogs, and humans. The 1918 Spanish flu, which caused a large pandemic, was found to be a type of bird flu (H1N1) that had adapted to infect humans [1, 2, 3, 4]. In April 2014, the first human case of H5N6 bird flu was found in Sichuan, China. Since then, many more cases have appeared in different parts of China. However, we still don't fully understand important details about how the disease spreads, its symptoms, and the risk of it passing from person to person [5, 6]. This study examines past cases of how the H5N6 bird flu spreads to humans, its epidemiology, genetic mutations, pathogenesis, and ways to treat and prevent it from becoming the next Disease X. The goal is to provide answers that can help better understand, manage and stop future outbreaks of H5N6.

Among the various avian influenza subtypes, H5N6 has emerged as a particularly concerning zoonotic pathogen. Since the first confirmed human case in Sichuan, China in 2014, the virus has caused nearly a hundred human infections, with a consistently high case fatality rate ranging from 52% to 61% depending of the study period and population examined [6, 7]. The majority of these cases occurred in southern China and were associated with direct or indirect contact with infected poultry, particularly in live bird markets or through backyard farming [8]. Epidemiological investigations show that exposure to sick or dead birds results in significantly higher mortality, likely due to greater viral loads [9]. Genetically, H5N6 viruses have undergone extensive reassortment, producing at least 13 distinct genotypes, and the predominant hemagglutinin subclade 2.3.4.4b is known to possess mutations such as Q226L and S227R in the HA gene that enhance binding to human‐type receptors [8, 10]. These genetic changes, coupled with high pathogenicity in animal models and limited but plausible evidence of human‐to‐human transmission, underscore H5N6's potential to breach species barriers. As such, H5N6 represents a credible candidate for the next “Disease X,” warranting urgent attention from both clinical and public health perspectives. This review aims to consolidate current knowledge of the virus's epidemiology, molecular evolution, and clinical behaviour to support future efforts in surveillance and preparedness in case of potential pandemics.

## 2 Epidemiology of H5N6 Infection

In 2014, a human case of H5N6 avian influenza came to world attention when it was found in Sichuan Province, China [11]. Since then, this virus has been primarily confined to China, with a few cases emerging in neighboring countries like Laos. There have been 93 laboratory‐confirmed human cases in the world as of October 2024. The infection has been particularly dangerous, with a case fatality rate (CFR) of 61 percent. The total death number among the infected remains 57. The recent‐most reported case is from China's Anhui province, detected on 17 June 2024 [7]. Most of these cases are due to direct or indirect contact with infected birds, most often at live bird markets and backyard poultry settings [11]. Table 1 gives an overview of the geographical distribution of H6N6 infections by region from 2014 to 2023 [6, 8, 9, 10, 11, 12, 13].

**Table 1:** Geographical distribution of H5N6 infections by region from 2014 To 2023.

Region/Province	Number of human cases	Sources
Guangxi Zhuang, China	19	[9, 11, 12]
Hunan, China	15	[9, 11, 12]
Guangdong, China	14	[6, 8, 9, 10]
Sichuan, China	13	[8, 9, 11, 12]
Jiangsu, China	5	[9, 11, 12]
Chongqing, China	3	[9, 11, 12]
Jiangxi, China	3	[9, 11, 12]
Anhui, China	2	[9, 11, 12]
Yunnan, China	2	[6, 8, 9, 10]
Fujian, China	2	[9, 11, 12]
Zhejiang, China	2	[8, 9, 10, 11]
Beijing, China	1	[9, 11, 12]
Guizhou, China	1	[9, 11, 12]
Hubei, China	1	[9, 11, 12]
Henan, China	1	[9, 11, 12]
Laos	1	[11, 13]
Vietnam	0	[12, 14]
South Korea	0	[12, 14]
Canada	0	[13, 14]

**Description:** The cited studies were used to extract key epidemiological details such as the number of cases and spread of the virus among different regions. The Table 1 summarizes the number of human infection cases of the virus in China and in neighboring countries like Laos and Vietnam for 2014–2023. According to data from several epidemiological studies, it places Guangxi, Hunan, and Guangdong as major hotspots of confirmed viral spread in animals. Guangxi Zuan and Hunan in China produced the highest number of human cases with 19,15 and 14 cases, respectively. Despite cases of animal infection, there were no human cases in places like Vietnam, South Korea, and Canada.

**Figure 1:**
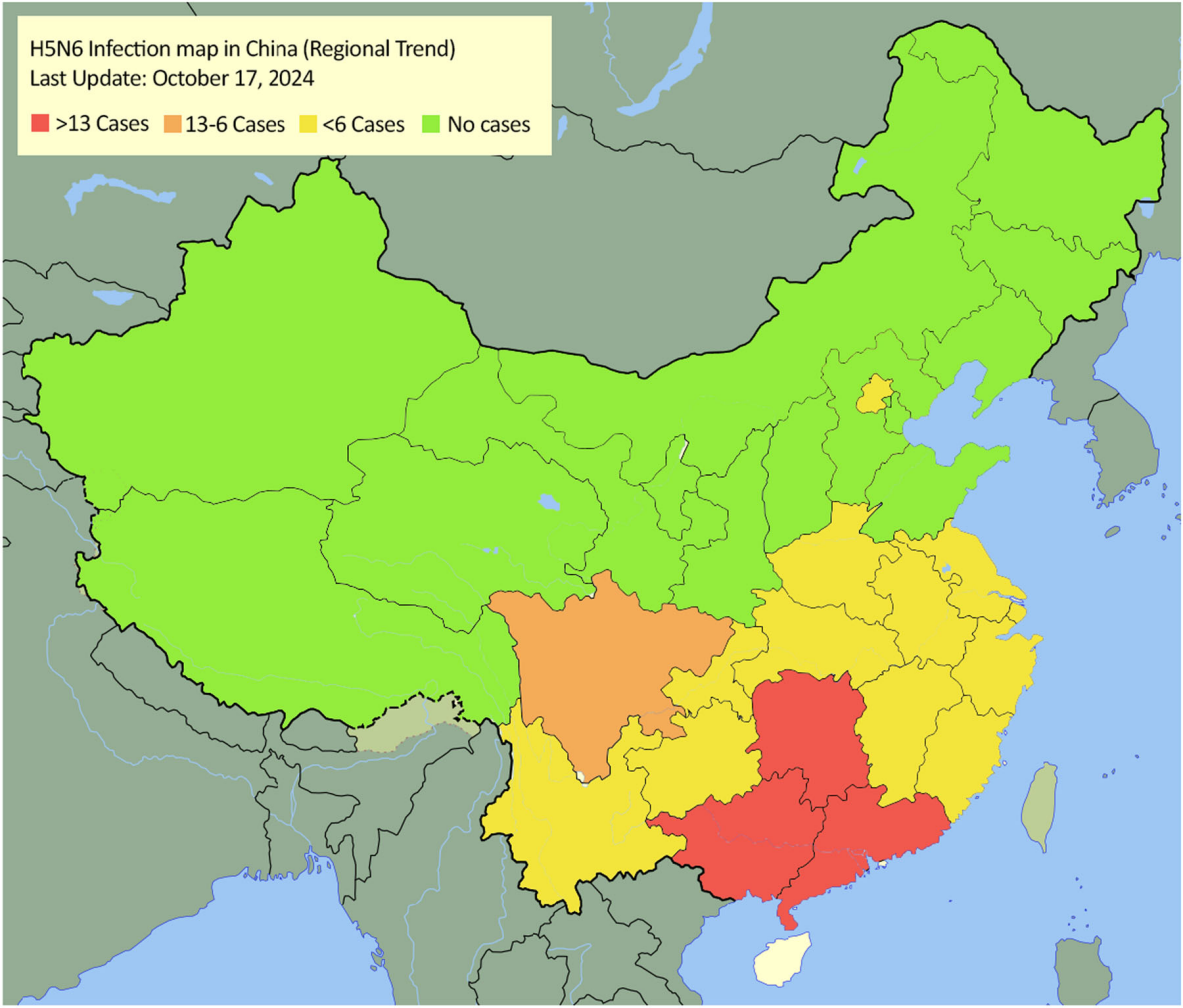
Map of China with the infected provinces highlighted based on severity of number of infections. The red area highlights provinces whose infection rate is higher than 13, orange highlights infection rate from 13 to 6, yellow highlights provinces with cases less than 6 and green denotes provinces free of H5N6 infection (as of 17th October, 2024). The map indicates that the highest infection rates occur in provinces that are located in the South‐east. As we go north, infection rate is reduced while the northern most provinces are completely free of H5N6 infection.

A Map of China showing the trend of human infection of H5N6 highlighted on the basis of provinces is illustrated in Figure 1 [6, 8, 9, 10, 11, 12, 13].

**Figure 2:**
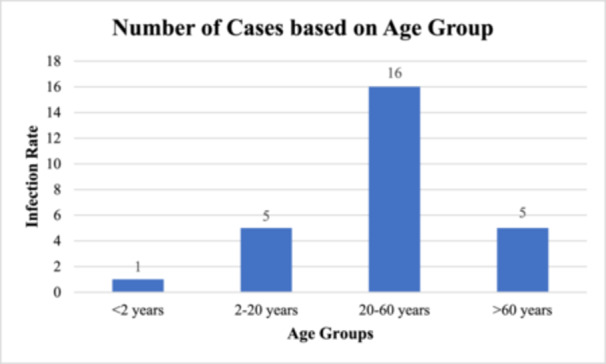
Number of H5N6 cases based on age groups. 16 cases (66.7%) are in 20–60 years age group. Amongst 5 cases are represented in the 2–20 years and > 60 years age groups, and the < 2 years group has only 1 case. The findings indicate that older people are more vulnerable to the virus and are thus at risk of severe outcomes.

Age distribution of H5N6 infected based on studies ranges from infants under 2 to 81 with a mean age of 30's or 40's. Most cases (66.7%) fall in the 20–60 age group [9, 13]. The virus has affected both males and females equally [9, 12, 13]. Higher risks of severe outcomes and mortality for older age (60 years and older) [9, 12]. Figure 2 illustrates the number of cases based on age groupings [9, 12, 13].

**Figure 3:**
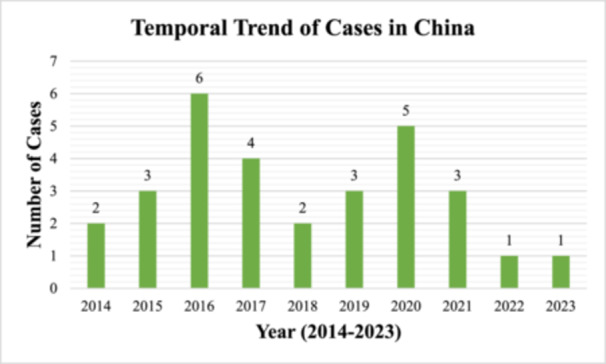
Temporal trend of H5N6 virus infections in China from 2014 to 2023. In 2016, there was a peak at 6 cases (25% of total cases during this period). In 2020, there was a slight decline before the virus resurged with 5 cases, almost certainly because new genotypes appeared. Subsequent years have lower infection numbers and only 1 case reported each for 2022 and 2023.

H5N6 has been able to jump from wild and domestic birds to humans because of the spread among them. These cases usually occur in cooler months as a result of increased contact with migratory birds and seasonal movement of poultry markets. In 2021, there were 37 cases reported, far higher than in any of the previous years. These cases were spread across 6 regions in China which differed from 1 to 5 regions per year in the previous years [11]. Surveillance systems erected during the COVID‐19 pandemic improved the detection and reporting of infections, leading to this uptick during the pandemic [7]. Although lower than 2021, human cases were still reported in 2023 suggesting that the virus is predominant in the region [11]. Figure 3 shows the temporal trend of the H5N6 virus in China [6, 9, 15].

Exposure data obtained from 71 cases indicate that 100% reported direct or indirect bird contact before infection. Other common exposure settings include visitation of live bird markets (LBMs), poultry farming or employment in the poultry industry, and direct or indirect contact with domestic poultry. Occupational data indicates that of 31 available cases, 71% reported employment in the poultry industry or the poultry market [11].

H5N6 hasn't achieved sustained human‐to‐human spread, but the risk it does pose remains. The ability of the virus to mix its genetic material with other influenza strains could place it on the fast track to becoming a more infectious virus. The detection of the virus in Canada within birds in 2022, for example, is a reminder that the virus remains relevant beyond Asia [11]. Vigilance remains vital, particularly in high‐risk environments, such as live bird markets or wet markets, where increased and enhanced biosecurity measures will go a long way to reducing the odds of H5N6 evolving into a much more serious public health threat.

## 3 Genetic Mutation of H5N6

Over time, the H5N6 avian influenza virus has undergone significant genetic changes which has been a cause of worry throughout the world. The virus has spread widely among wild birds, poultry, and in rare cases humans, especially by clade 2.3.4.4. H5N6 was first identified in 2014, and since then has evolved into a variety of subtypes, adapting in different ways. Other subclades include 2.3.4.4b, which have been linked to outbreaks in Korea and China, for instance [16, 17].

From 2019 to 2020 the virus was found in wild birds and live poultry markets in Bangladesh. Studies suggest that migratory birds likely brought the virus from Central Asia. The strain found in Bangladesh—of subclade 2.3.4.4 h—is genetically related to virus strains that was previously found in China and Mongolia [16]. What's worrying is that the virus was found in seemingly healthy wild and domestic birds, demonstrating how easily the disease can spread without obvious signs and symptoms of illness. A major outbreak in 2020 in China's swans in Xinjiang showed just how dangerous the virus could be to birds. Genetic analysis revealed that these viruses turned out to have a high degree of similarity to ones from Guangdong Province, which confirmed how fast the virus can spread across a region [18]. The hemagglutinin (HA) and neuraminidase (NA) genes of these strains are mutated, making them highly pathogenic in birds and potentially more dangerous in mammals, although less well‐defined in humans [18].

In South Korea, meanwhile, scientists found new versions of H5N6 in 2023 and have found that the virus continues to evolve. What is particularly notable about these new strains is that they represent the genetic combination of H5N1 and low pathogenicity viruses, that show that the virus can interchange genetic material with others [17]. The constant evolution of the virus means that it is harder to predict how it may behave in the future, thus posing a serious risk that it will evolve into a more dangerous version. The virus is a rapidly changing strain that isn't only a threat to birds but could also be a threat to humans. Its adaptability and ability to spread makes this a virus that needs to be watched closely, and continued research into [16, 17].

Genomic analysis of H5N6 viruses isolated from poultry markets in Guangdong based on GISAID sequences, revealed several important mutations and reassortment events. Most isolates belonged to clade 2.3.4.4b, which replaced earlier clades such as 2.3.4.4 h. A notable mutation observed in many H5N6 isolates was an 11‐amino acid deletion in the stalk region of the N6 neuraminidase gene, a change associated with increased virulence and replication efficiency in poultry. In addition, full‐genome analysis identified extensive reassortment involving internal gene segments, particularly PB1 and PA, derived from both highly pathogenic and low pathogenic avian influenza viruses. While known mammalian‐adaptive mutations such as PB2‐E627K and PB2‐D701N were not detected, the recurrent genetic reshuffling and expansion of genotypes indicate an ongoing diversification that may facilitate future cross‐species transmission. Mutations such as S137A, S158N, and T160A in the HA gene, which increase human receptor binding affinity, were universally present, highlighting the growing zoonotic potential of these evolving H5N6 strains [10].

## 4 Transmission and Spread of H5N6 to Cause Infection

Avian flu virus H5N6 mostly spreads from bird to bird via droppings, secretions, and contaminated environments [11, 14]. The virus is the natural host of waterfowl but can be spread to humans through close contact with infected poultry. Such activities include bird handling at live markets, in slaughterhouses, or in contact with domestic bird populations [14].

H5N6 in the past has spread to several continents, with outbreaks now reported in Asia, Europe, and Africa. Of the 85 confirmed human cases, 84 were in China with one case in Laos by June 2023. Most people had been exposed to an infected bird before becoming ill with 84 percent reporting direct bird contact [11]. Although experts say that human‐to‐human transmission is still very rare, they fear the virus could mutate and spread more easily between people [14].

Live bird markets and agricultural activities are linked to the virus's continuing spread. The death rate due to human infections is around 61%. Infection can develop at any time of year, but cases are slightly down in summer [11]. Avian flu viruses that circulate the world—including H5N6—have a greater chance of changing in ways that could make them more dangerous to humans [14].

H5N6 persists in wild bird populations and domestic birds, so further outbreaks and human cases are expected. Continued surveillance and enhanced biosecurity measures are necessary to stop the virus and future infections [11, 14].

## 5 Disease Pathogenesis of H5N6 Infection in Humans

The H5N6 avian influenza virus has become a growing concern due to its severe impact on human health with a case fatality rate ranging between 44% and 55.4% as reported in some studies [8, 14] while other studies report a mortality rate as high as 61% [11].

Table 2 classifies mortality rates based on criteria like age group, exposure type, gender, and geographic region [8, 9, 11, 13, 14].

**Table 2:** Classification of mortality rates.

Criteria	Subcategories	Sources
Age Group	< 2 years: 100%, 2–20 years: 50%, 20–60 years: 37.5%, > 60 years: 100%	[9, 12, 14]
Exposure Type	Sick/Dead Birds: 100%, Live Birds: 35.3%, Slaughtered Birds: 8.3%	[9, 12]
Gender	Male: 41.7%, Female: 63.6%	[9, 15]
Geographic Region	Guangdong: Higher mortality, Guangxi: Higher exposure rate, Sichuan: Moderate risk	[8, 9, 12]
Underlying Health Conditions	Higher mortality in older adults with underlying conditions like respiratory issues	[8, 9, 14]

**Figure 4:**
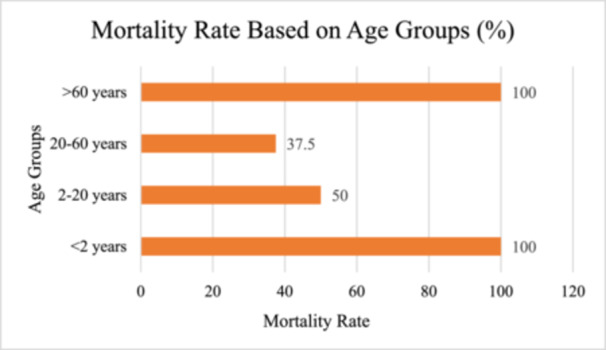
H5N6 infections cause highest mortality rates in the < 2 years and > 60 years age groups, both with 100% fatality rate. Mortality rate is 37.5% for the 20–60 age group, which is the majority, and the mortality rate for the 2–20 age group is 50%. In these findings, these individuals are also especially vulnerable to severe outcomes.

A graphical representation of the mortality rate based on different age groups is shown in Figure 4 [9, 12, 13].

Fever and cough are common symptoms, but most patients require intensive care soon afterwards with pneumonia. About 93.8 percent of those infected wind up in the ICU and often require mechanical ventilation or, in the worst cases, lifesaving interventions such as extracorporeal membrane [8]. Other symptoms include upper respiratory symptoms and muscle aches (myalgia). The infection quickly began to spread to lower respiratory tract diseases such as pneumonia, acute respiratory distress syndrome (ARDS), multiorgan failure, and in the severest cases death [11].

There has been no consistent evidence of human to human transmission, but occasional cases within households suggest it could be possible under some circumstances [8, 14]. Older adults and people with certain underlying conditions – such as heart or lung disease—have generally been at greater risk of higher fatality rates [15]. From symptom onset to hospitalization, the median delay was 4 days (range: 0‐13 days) [11].

Long‐term consequences of the disease have been identified with severe and irreversible sequelae in some patients being reported such a case of a Zhejiang patient with pulmonary fibrosis and lung damage that caused a severely impaired quality of life, including chronic pulmonary bleeding and a Jiangsu patient with encephalitis who developed follow‐up neurological complications, including posterior cerebral atrophy [9].

The biggest problem with H5N6 is its ability to evolve. The virus has undergone genetic changes that have allowed it to infect both birds and humans [15]. Early onset treatment is important, and delays in treatment with antiviral therapies are associated with poorer outcomes [8]. Meanwhile, of the cases for which there was known disposition, 90 percent were in critical condition at the most recent report [11].

## 6 Therapeutic and Preventive Measures of H5N6 Infection

H5N6 may be prevented by avoiding exposure to the virus. It includes avoiding close contact (direct) with live or dead poultry in areas where there are known outbreaks [19, 20]. People who work with birds or those who are at risk with exposure should wear protective gear such as gloves, masks and eye protection [21]. Washing hands thoroughly after any direct contact with birds is a must [20].

Along with killing the virus, cooking poultry thoroughly (at least 165 °F, or 74°C) makes the meat safe to eat [19]. For anyone going to countries with H5N6 cases, it is advisable to stay away from places like live poultry markets [20]. Reporting any sick or dead birds to local authorities and keeping up to date with safety guidelines can do a great deal in protecting the individual and their community from the risk of H5N6 [19, 21].

Antiviral medications such as oseltamivir, zanamivir, and peramivir will treat the infection. Ideally, these drugs should be taken initially 48 h after the first symptom. The illness may be less severe and shorter in duration if the medications are taken early. For those with more serious cases (pneumonia, difficulty in breathing) support care (oxygen therapy, fluids, etc) is key [19, 21]. This care may also include the use of ventilators to help with breathing [20]. How quickly the treatment process starts and in how good shape the patient is playing a large role in whether or not these treatments will be successful.

No specific vaccine is currently approved to protect people against H5N6. There are some experimental vaccines developed, but they have not been widely available [21]. Because H5N6 is so unique, seasonal flu vaccines, updated to tackle new strains, don't always work against it [19]. Future outbreaks are in mind, and researchers are working on developing more targeted vaccines [20]. The unavailability of a vaccine for the virus indicates our unpreparedness for a potential mutation of the strain and its eventual evolution towards becoming a pandemic.

Primarily, inactivated vaccines targeted at specific strains of avian influenza viruses are vaccines developed for poultry against the H5N6 virus. Table 3 lists the types of vaccines used for poultry and in which region they are largely used in [9, 10, 11, 14].

**Table 3:** Poultry vaccines used against H5N6.

Vaccine Type	Description	Used In	Source
H5 Reassortant Inactivated Vaccines (Re‐11, Re‐12)	They contain H5N6 hemagglutinin gene and internal genes that came from other avian influenza viruses.	China (Guangdong, Guangxi)	[9]
Bivalent H5/H7 Vaccines	Specifically target multiple avian influenza subtypes particularly subtypes H5 and H7.	China	[8, 15]
Oil‐Emulsion Inactivated Vaccines	They are inactivated vaccine prepared with oil‐based adjuvant to evoke better immune response.	China, large‐scale poultry farms	[6]

Table 4 lists some candidate vaccines that are under trial for H5N6 [21, 23].

**Table 4:** Vaccine candidates in development for H5N6.

Vaccine candidate	Type	Immune response	Virus subtypes	Sources
H5‐Re13, H5‐Re14, H7‐Re4	Inactivated virus vaccines	Antibody‐mediated response	H5N1, H5N6, H5N8, H7N9	[21, 22]
MF59‐adjuvanted seasonal influenza vaccine (Fluad®)	Trivalent inactivated vaccine	Antibody and cell‐mediated response	H5N1 (potential for H5N6)	
AS03‐adjuvanted prepandemic H5N1 vaccine	Inactivated virus vaccine	Antibody and cell‐mediated response	H5N1 (potential for H5N6)	
Newcastle Disease Virus (NDV) H5 Vaccine	Vector‐based vaccine	Antibody, mucosal, and cell‐mediated response	H5N1 (potential for H5N6)	

## 7 Prediction of H5N6 Infection to Cause Global Pandemics

In 2021, when human infections with the H5N6 virus spiked, researchers singled out new genotypes known as 2021 A, 2021B, 2021 C, and 2021D. In these findings, we also see that the virus is still evolving genetically, in particular its reassortment with clade 2.3.4.4b strains. This shows the ability of the virus to cross species boundaries, continuing to acquire genes from other viruses of avian influenza H5N8 and H9N2. The changes help the virus adapt better to bird and mammal hosts, increasing its ability to transmit from one species to the next. [8] H5N6 is of special concern because, unlike most avian influenza viruses, it has evolved to bind to human respiratory cell receptors. As opposed to typical avian influenza viruses, which mainly bind to avian‐type receptors (SAα2,3 Gal), H5N6 can also bind to human‐type receptors (SAα2,6 Gal). That means the virus is becoming more adept at infecting human respiratory tissues. Research in ferret models, which are typically used to study human‐to‐human flu transmission, has found that H5N6 can infect ferrets through direct contact and therefore may become optimized for human‐to‐human transmission [12, 23].

H5N6 is one of a group of H5 viruses transmitted by migratory birds and via the poultry trade across Asia, Europe, and beyond. The spread of this virus around the world means there is higher potential of humans’ exposure and more opportunities for the virus to develop and spread more easily between humans. The virus is evolving in a manner that bestows concern for what it may achieve in terms of a more rapid transmission capability in the future [12, 13]. H5N6 has not yet shown airborne transmission between humans, a key determinant of pandemic potential, but experiments in ferrets show that the virus is capable of being passed via direct contact. Although it hasn't reached the level of easy human‐to‐human spread, H5N6 has shown a worrying ability to infect mammals more effectively than earlier strains like H5N1 [13, 23]. H5N6 has replaced H5N1 as the predominating avian influenza in southern China, occurring mainly in ducks and geese [12]. The absence of specific antiviral drugs and vaccines, along with the need for ongoing research, stresses the importance of being prepared and staying alert. To prevent a potential pandemic, we must track genetic changes, understand transmission patterns, and develop treatments and vaccines. This will need global cooperation and continuous research [7]. H5N6's ability to bind to human respiratory cell receptors marks a significant shift, allowing it to potentially infect human tissues more effectively and raising alarms about its pandemic risk. As H5N6 spreads globally through migratory birds and poultry trade, the chances of human exposure increase, presenting more opportunities for the virus to evolve into a more easily transmissible form. To effectively combat the rising threat of H5N6, it is crucial to enhance global surveillance and foster collaborative research efforts. By doing so, we can better understand its evolution and transmission, ultimately safeguarding public health against future pandemics.

## 8 Conclusion

H5N6 avian influenza is a serious public health concern because of its high mortality rate, broad adaptability, and ability to infect humans. While the virus hasn't yet spread easily between people, experts worry about how far it will evolve and whether it can continue to bind to human respiratory cells before it can be contained as a pandemic. This paper emphasizes the need for continually looking, particularly in high‐risk areas such as live poultry markets, and the need for stronger biosecurity. They also line up accelerating vaccine development and international cooperation as essential tactics for narrowing the gap to when the H5N6 might become the next Disease X. In light of the dangers that new infectious diseases will pose to the world, and an increased appreciation of the need to be prepared for emerging viruses like H5N6, there is a need to be ready for worse to come. So, it may be proactive to take measures now to help save lives and minimize the repercussions of this rapidly morphing virus.

## Author Contributions

**Shoubeho Sadique Shandhi:** Writing – original draft; Writing – review and editing; Software. **Mosiur Rahman:** Writing – review and editing; Writing – original draft. **Mohammad Shahriar:** Conceptualization; Supervision; Writing – review and editing. **Ramisa Anjum:** Conceptualization; Supervision; Writing – review and editing.

## Ethics Statement

The authors have nothing to report.

## Conflicts of Interest

The authors declare no conflict of interest.

## 1 Transparency Statement

The lead author Ramisa Anjum affirms that this manuscript is an honest, accurate, and transparent account of the study being reported; that no important aspects of the study have been omitted; and that any discrepancies from the study as planned (and, if relevant, registered) have been explained.

## Data Availability

Data sharing not applicable to this article as no datasets were generated or analysed during the current study. All authors have read and approved the final version of the manuscript. Ramisa Anjum had full access to all of the data in this study and takes complete responsibility for the integrity of the data and the accuracy of the data analysis The authors confirm that the data supporting the findings of this study are available within the article [and/or] its supporting materials.
